# Factors influencing preoperative stress response in coronary artery bypass graft patients

**DOI:** 10.1186/1471-2253-4-7

**Published:** 2004-09-23

**Authors:** Astrid M Morin, Götz Geldner, Udo Schwarz, Martin Kahl, Hans A Adams, Hinnerk Wulf, Leopold HJ Eberhart

**Affiliations:** 1 Department of Anaesthesiology and Critical Care Medicine (Professor and Chairman: Hinnerk Wulf) Philipps-University Marburg Baldingerstrasse 35043 Marburg Germany

## Abstract

**Background:**

In many studies investigating measures to attenuate the hemodynamic and humoral stress response during induction of anaesthesia, primary attention was paid to the period of endotracheal intubation since it has been shown that even short-lasting sympathetic cardiovascular stimulation may have detrimental effects on patients with coronary artery disease. The aim of this analysis was, however, to identify the influencing factors on high catecholamine levels *before *induction of anaesthesia.

**Methods:**

Various potential risk factors that could impact the humoral stress response before induction of anaesthesia were recorded in 84 males undergoing coronary aortic bypass surgery, and were entered into a stepwise linear regression analysis. The plasma level of norepinephrine measured immediately after radial artery canulation was chosen as a surrogate marker for the humoral stress response, and it was used as the dependent variable in the regression model. Accordingly, the mean arterial blood pressure, heart rate and the calculated pressure-rate product were taken as parameters of the hemodynamic situation.

**Results:**

Stepwise regression analysis revealed that the oral administration of low-dose clonidine (mean dose 1.75 μg·kg^-1^) on the morning of surgery was the only significant predictor (p = 0.004) of the high variation in preoperative norepinephrine plasma levels. This intervention decreased norepinephrine levels by more than 40% compared to no clonidine administration, from 1.26 to 0.75 nmol·l^-1^. There was no evidence for dose-responsiveness of clonidine. All other potential predictors were removed from the model as insignificant (p > 0.05). The use of beta-blocker, ace-inhibitors, ejection fraction, and body mass index were significant determinants for the hemodynamic situation (heart rate, mean arterial pressure, pressure rate product) of the patient during the pre-induction period.

**Conclusion:**

The oral administration of clonidine is the only significant predictor for the observed variation of norepinephrine levels during the preoperative period. Lack of significant dose responsiveness suggests that even a low dose of the drug can attenuate the preoperative stress response and thus is recommended in cardiovascular high risk patients.

## Background

There is increasing evidence that sympathetic nervous system mediated cardiovascular stimulation with increased catecholamine blood levels is the principal mechanism responsible for perioperative tachycardia and hypertension, myocardial ischemia and infarction [1-3]. Even short-lived changes may have detrimental effects on the coronary circulation of high-risk patients, with higher rates of morbidity and lethality [4,5]. Thus, many studies have concentrated on the stressful stimulus of endotracheal intubation, and a number of pharmacological attempts have been used to attenuate the hemodynamic response, including the use of high doses of opioids, α_2_-adrenergic receptor agonists, β-adrenergic blocking drugs or other antihypertensive drugs [3,4,6-19].

However, little attention has been paid to the stress response of cardiac high risk patients when entering the operating area, during initiation of routine monitoring, and finally during awake venous and arterial canulation. Especially the latter procedure can cause significant discomfort for patients even when performed under local anaesthesia [20]. In several trials a high inter-individual variation of pre-induction norepinephrine levels, heart rate and blood pressure could be noticed [21]. Thus, this observational analysis was performed in patients undergoing coronary aortic bypass surgery (CABG-surgery) in order to identify patients at high risk for increased sympathoadrenergic stress response during the immediate preoperative period using norepinephrine levels and the hemodynamic status as surrogate measures.

## Methods

After Ethics Committee approval was obtained, patients gave their written and informed consent. Eighty-four consecutive male patients undergoing CABG-surgery were enrolled into this observational study. The only exclusion criterion was emergency operation.

**Figure 1:**
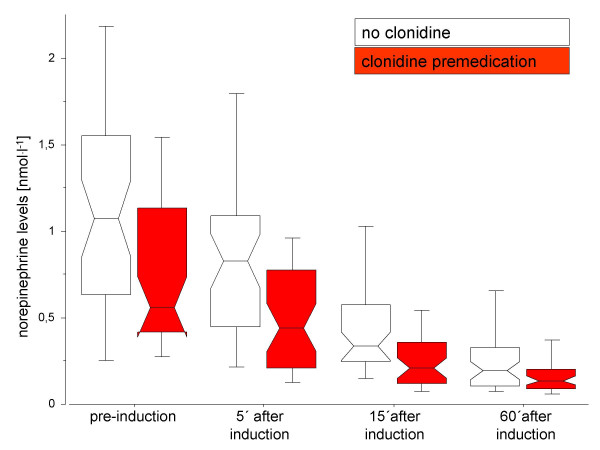
Norepinephrine levels in patients with and without clonidine premedication.

Due to the observational character of the study, drugs administered preoperatively (including clonidine) were given at the discretion of the anaesthetist performing the preoperative examination. Each patient received an oral premedication with clorazepate 20 mg in the evening before surgery and in the morning of surgery. In about half of the patients, benzodiazepine premedication was combined with clonidine 75–300 μg. Patients were maintained on their regular cardiac and antihypertensive medication up to the day of surgery but all inhibitors of platelet aggregation were discontinued 3–7 day preoperatively. After arrival at the operating theatre an i.v. line was initiated and 500 ml hydroxyethyl-starch (10%, 200000 Dalton) was infused. A 12-lead channel ECG with an automatic ST-segment analysis, oxygen saturation and invasive blood pressure monitoring were connected to the patients. A radial artery catheter was then inserted after local anaesthesia with 1 ml mepivacaine 1%. Heart rate (HR) and mean arterial blood pressure (MAP) were recorded every 15 seconds online using a Laptop computer connected to a Solar 9500 monitor (General Electrics, USA). HR and MAP were multiplied to receive the pressure-rate product (PRP). These variables were used as a measure of the hemodynamic stress response. To determine humoral stress response, an arterial blood sample for the measurement of norepinephrine plasma level was taken immediately after placing the radial artery catheter (this measurement was the main outcome of the study) and 5, 15, and 60 minutes after endotracheal intubation was performed (these measurements were used in an additional explorative analysis, see figure 1). 10 ml plastic lithium-heparin tubes were used for this purpose. Specimens were placed on ice immediately after sampling, spun in a centrifuge for 20 minutes and plasma was separated and stored at -70°C pending analysis. Plasma norepinephrine levels were determined by high-performance liquid chromatography (HPLC) with electrochemical detection (Millipore, Billerica, Mass. USA). The lower limit of detection for norepinephrine was 0.018–0.024 nmol·l^-1 ^and the same-day coefficient of variation for norepinephrine measurements determined by repeated measures of a standardized probe was 3%.

General anaesthesia was standardized. After administration of midazolam 0.05 mg·kg^-1 ^and a three minute period of preoxygenation, anaesthesia was induced using a continuous infusion of propofol (10 mg·kg^-1.^h^-1^) and sufentanil (10 μg.kg^-1.^h^-1^). After loss of consciousness propofol was reduced to 3 mg·kg^-1.^h^-1 ^and sufentanil to 1.5 μg·kg^-1.^h^-1^. Endotracheal intubation was performed after administration of pancuronium bromide 0.1 mg·kg^-1^.

To allow comprehensive analysis of potential factors associated with a reduced stress response, the following data were recorded prospectively:

• Age

• Bodyweight

• Height

• Body mass index (BMI)

• Clorazepate dose per kilogram bodyweight

• Clonidine (yes – no)

• Clonidine per kilogram bodyweight

• Time from morning premedication until observational period

• Inhibitors of the angiotensine converting enzyme system (ace-inhibitors)

• Beta-blocking drugs

• Calcium antagonists

• Angiotensin-2 receptor inhibitors

• Left ventricular ejection fraction (EF)

• Number of affected vessels

### Statistical analysis

A power analysis had revealed that 80 patients provide a power of more than 95% to detect an R^2 ^of 0.3 and higher, attributed to 14 independent variables using an F-test with a significance level of 0.05. All potential relevant factors were subjected to a stepwise linear regression analysis using a backward technique. In each step the least significant factor was eliminated when F was lower than 3.96. The quality of the regression model was judged using the Durbin-Watson statistic (a value between 0 and 4 indicating the amount of autocorrelation within the model with an optimum of 2.0), and by checking if the standardized residuals are normally distributed. All calculations were performed using SPSS 11.0 for Windows. All continuous data are presented as mean and standard deviation when normally distributed and as median (25^th^–75^th ^percentile) when normal distribution had to be rejected using the Kolmogorov-Smirnov-test.

## Results

Stepwise regression analysis revealed that the single administration of low-dose clonidine (mean dose 1.75 μg·kg^-1^) on the morning of surgery was the only significant predictor (p = 0.004) of the high variation in preoperative norepinephrine plasma levels. This intervention decreased norepinephrine levels by 40% compared to no clonidine administration (from 1.26 to 0.75 nmol·l^-1^). In this analysis, the dichotomous variable (clonidine administration: yes-no) was a better predictor for the norepinephrine levels than variables including the clonidine dose (absolute dose or dose per body weight), indicating that our data provide no evidence for a strong dose-responsiveness of clonidine in this context.

All other of the investigated factors (see methods) were removed from the regression model as not significant. The two factors that were eliminated with a p < 0.1 during the last but one and during the final step were body mass index (removed in step 11 with a p = 0.064) and age (removed in step 12 with a p = 0.076). Both factors were associated with increased norepinephrine levels.

The overall quality of the regression model was excellent. The Durbin-Watson coefficient was 2.04 (very near to the optimum of 2.0) and the standardized residuals were normally distributed.

For mean arterial blood pressure, heart rate and the calculated pressure rate product, however, preoperative clonidine administration was not an influencing factor.

For the mean arterial pressure (MAP), a higher ejection fraction (EF) was a statistically significant predictor (p = 0.024). Each 10% increase of EF was associated with a 2.7 mmHg higher MAP. Administration of an ace-inhibitor was the second predictor in the final model of MAP (p = 0.03). These patients had a 7.5 mmHg lower MAP than patients without ace-inhibitors.

For heart rate (HR) there were three significant predictors that remained in the model. Administration of beta-blockers and ace-inhibitors were both associated with a decreased HR (p = 0.004). Each of them decreased HR between 6–7 beats per minute (bpm). Additionally, a higher BMI was associated with a 1.3 bpm higher HR per kg·m^-2 ^(p = 0.001).

Since the PRP is the product of HR and MAP, it is not surprising that similar variables contributed to its prediction. These were the administration of beta-blockers (p = 0.017) and ace-inhibitors (p = 0.004), each of them reducing the PRP, whereas the EF was associated with an increase in PRP (p = 0.014).

No patient had signs of cardiac ischemia on arrival at the operating theatre until induction of anaesthesia (defined as ST-T change > 0.1 mV in any ECG lead). There were no major adverse events during the entire induction period and surgery.

## Discussion

The α_2_-adrenergic receptor agonist clonidine acts by decreasing central sympathetic nervous system activity in all hyperadrenergic situations. In addition to its sedative, anxiolytic, analgesic and antihypertensive properties [6,22] it has shown to improve congestive heart failure, to optimize the myocardial oxygen supply / demand ratio in ischemic heart disease [23,24] and to reduce attacks of angina pectoris [25,26].

In many investigations attention has been drawn to the stressful stimulus of endotracheal intubation [3,4,6-19], as it has been shown that even short-lasting sympathetic cardiovascular stimulation may have detrimental effects on the coronary circulation of patients with coronary artery disease (CAD), with higher rates of morbidity and lethality [4,5], However, little emphasis has been paid to the preoperative period where patients may be stressed by or because of the upcoming procedure. Furthermore, transfer to the operating theatre, initiation of routine monitoring, and venous and arterial canulations are stressors for the patients. In this context it is certainly a drawback of our study that we did not record the level of preoperative sedation or anxiolysis using clinical measurements on appropriate scales. Instead of this, only a rough judgment was made (awake versus asleep but rousable) that did not allow a valid analysis of the data. Previous data, however, have shown potent anxiolytic and sedative properties of the drug [27].

Thus, it was the major aim of this observational study to identify factors that might contribute either to increased humoral stress or that might help to attenuate this response.

Our results show, that a single application of low dose oral clonidine was the only factor that was associated with significantly decreased norepinephrine levels on arrival at the pre-induction area. The question that arises from this observation is, if this is simply an association (or statistically spoken a co-linearity between other protective factors) or if clonidine premedication is the cause for lower norepinephrine levels. In our opinion the latter is the case. Firstly, there were no differences considering any other variables between those patients who had received clonidine and those who had not (see table 1). Thus, it is unlikely that other factors were responsible for the reduced norepinephrine levels. Secondly, there is good evidence from the literature that clonidine is a powerful drug that attenuates stress response of various causes [3,4,6-19].

**Table 1:** Patients' demographic data and preoperative condition. Data are presented for all 84 patients that were included in the study, and separately for those patients receiving clonidine and those without oral clonidine premedication. Values are expressed as mean ± standard deviation, median (25^th^–75^th ^percentile), or n = (%).

	All patients	Patients with clonidine morning premedication	Patients without clonidine morning premedication
	n = 84	n = 42	n = 42
Age (years)	66 ± 9	65 ± 9	67 ± 8
Bodyweight (kg)	82 ± 10	82 ± 11	82 ± 10
Height (cm)	173 ± 6	173 ± 6	173 ± 5
BMI (kg · m^-2^)	27.3 ± 3.1	27.3 ± 3.2	27.4 ± 3.0
EF (%)	62 ± 14	61 ± 13	63 ± 16
Affected vessels (n = / %)			
n = 1	3 (4%)	0 (0%)	3 (7%)
n = 2	16 (19%)	9 (21%)	7 (17%)
n = 3	64 (76%)	33 (79%)	31 (74%)
n = 4	1 (1%)	0 (0%)	1 (2%)
Pre-treated with (n= / %)			
ace-inhibitors	43 (51%)	17 (40%)	26 (62%)
Beta-blockers	55 (65%)	26 (62%)	29 (69%)
Calcium-antagonists	12 (14%)	5 (12%)	7 (17%)
Clonidine dose (n= / %)			
75 μg		8 (19%)	
150 μg	n/a	33 (79%)	n/a
300 μg		1 (2%)	
Time from morning premedication until observational period (hours)	1.0 (0.5–4.5)	2.5 (0.5–5.0)	1.0 (0.5–4.5)
Heart rate [bpm]	66 ± 11	66 ± 10	66 ± 12
Mean arterial blood pressure [mmHg]	102 ± 16	100 ± 15	104 ± 17
Pressure rate product [mmHg·bpm]	6750 ± 1640	6660 ± 1600	6850 ± 1690
Plasma norepinephrine level (nmol·l^-1^)	1.00 ± 0.82	0.75 ± 0.48	1.26 ± 1.00

However, it is interesting to notice that the mean dose administered to our patients (1.75 μg·kg^-1^) was low compared to all other trials. Data concerning the appropriate dose of clonidine to attenuate the stress response to intubation vary considerably between 0.625 and 10 μg·kg^-1^. For example, one trial demonstrated that clonidine 0.625 and 1.25 μg·kg^-1 ^i.v. were sufficient to attenuate pressure response to laryngoscopy and intubation [28], whereas in another one [19] evaluating the dose-response effects to laryngoscopy and intubation, 2 μg·kg^-1 ^clonidine i.v. was equally effective as placebo, and only 4 and 6 μg·kg^-1 ^significantly attenuated hemodynamic and adrenergic reactions in an equal manner. It could also be shown that 4 or 6 μg·kg^-1 ^were necessary to reduce norepinephrine levels *before *induction of anaesthesia, however 2 μg·kg^-1 ^where not sufficient in this setting [19].

In our trial as well as in all other studies with even much higher doses, clonidine was well tolerated and did not produce any adverse hemodynamic effects.

In our analysis there was no strong evidence for a dose responsiveness of orally administered clonidine. First, in the regression model catecholamine levels could better be predicted by the dichotomous variable and second, there was only a weak correlation between the weight adjusted clonidine dose on the one hand and norepinephrine levels on the other hand (Pearson's correlation coefficient was -0.31, Spearman's rank correlation coefficient was -0.30). Furthermore, a post-hoc comparison between the patients receiving either 75 or 150μg clonidine did not show relevant differences (p = 0.91 using the Mann-Whitney U-test).

Higher age and higher body mass index showed a non-significant tendency to increase the catecholamine concentration. No other of the investigated factors (body weight, height, time from morning premedication until observational period, benzodiazepine dose per kilogram bodyweight, ace-inhibitors, beta-blocking drugs, calcium antagonists, EF, number of affected vessels) had statistically significant impact on norepinephrine levels.

An explorative post-hoc analysis of the impact of clonidine premedication (none versus any dose) and clonidine dose on norepinephrine levels during the entire induction period proves the results of the main analysis. There was a pronounced reduction of norepinephrine plasma levels after induction of general anaesthesia with lower values in the clonidine-group. However, a statistically significant interaction term (p = 0.012) suggests that the fall of norepinephrine levels are more marked in the untreated group and thus mainly caused by induction of general anaesthesia rather than effects of clonidine (figure 1).

## Conclusion

This observational trial demonstrates that patients undergoing coronary artery bypass graft surgery have a great variation of norepinephrine levels when entering the operating theatre. We could identify oral clonidine premedication as the only predictor for increased humoral stress response. There was no strong evidence for a dose dependency, indicating that even small doses, like 75–150 μg attenuate the humoral stress response before coronary artery bypass graft surgery. Clonidine did not have a negative impact on hemodynamic parameters.

## Competing interests

None declared.

## Authors' contributions

AMM processed the data and wrote the manuscript.

GG conceived the study, collected the clinical data and participated in its design.

US collected the clinical data.

MK designed the study and collected the clinical data.

HAA performed the laboratory investigations.

HW participated in the conception of the study.

LHJE designed the study, performed the statistical analysis and extensively revised the manuscript.

All authors read and approved the final manuscript.

## Pre-publication history

The pre-publication history for this paper can be accessed here:


